# Neuroendocrine Adenoma of Middle Ear Causing Acute Onset Facial Palsy- A Rare Case Report

**Published:** 2019-09

**Authors:** Shakeel-Uz Zaman, Iqra Zakir, Qazi Faraz, Amal-Asif Ahmed, Praneta Kulloo, Shakil Aqil

**Affiliations:** 1 *Department of Otorhinolaryngology-Head and Neck Surgery, Liaquat College of Medicine & Dentistry and Darul Sehat Hospital, Karachi, Pakistan.*; 2 *Department of Otorhinolaryngology-Head and Neck Surgery, Liaquat National Hospital & Medical College, Karachi, Pakistan.*; 3 *Department of Otolaryngology-Head and Neck Surgery, St Mary's Hospital, London, United Kingdom.*

**Keywords:** Acute, Adenoma, Facial nerve, Middle ear, Neuroendocrine, Palsy

## Abstract

**Introduction:**

Acute facial nerve palsy secondary to neuroendocrine adenoma of the middle ear (NAME) is a rare disorder. There is only one case report in the literature describing similar findings.

**Case Report:**

A 50-year-old man initially presented to ENT clinic with a right-sided middle ear mass and normal facial nerve function. Over the next six days, he developed House-Brackmann grade II facial paralysis. He underwent urgent surgical exploration of the tympanic cavity and excision of the middle ear mass via a post-auricular approach. Histopathological and immunohistochemical analysis revealed NAME. Three weeks after the surgery, facial nerve function returned to normal. No recurrence was found at a 3-year follow-up.

**Conclusion:**

Acute onset facial palsy induced by NAME is an extremely rare disorder. For a patient already affected by hearing impairment resulted from middle ear mass, facial weakness can have a significant additional detrimental impact on their wellbeing. The early complete excision of tumor is recommended not only as a curative treatment but also restoration of facial function.

## Introduction

Neuroendocrine adenoma of the middle ear (NAME) is an uncommon benign neoplasm of the middle ear, first reported by Hyams et al. and Derlacki et al. in 1976 (1,2). These tumors exhibit dual differentiation in the form of exocrine epithelial and neuroendocrine components (3-5). The diagnosis of these tumors is a challenging issue given their asymptomatic features. Facial paralysis secondary to these masses is rare (6,7). The early recognition and treatment of NAME are important to reduce the complications of an expanding mass (e.g., permanent facial palsy and hearing loss) which can have a significant impact on the patient’s wellbeing. Herein, we reported a rare case of acute facial palsy resulting from NAME and recovering completely after the urgent surgical excision. Moreover, we also reviewed the current literature and briefly discussed the clinical presentation, radiological findings, histopathology and treatment of NAME. 

## Case Report

**Fig 1:**
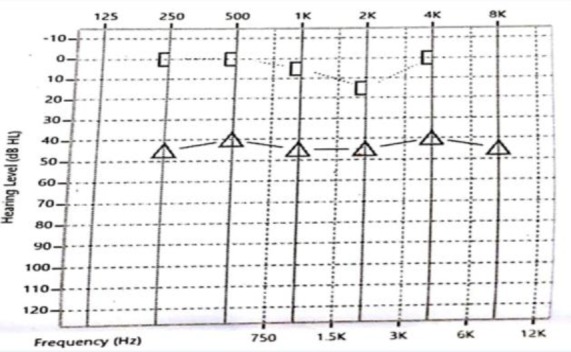
Pre-operative pure tone audiogram of right ear demonstrating conductive deafness

A 50-year-old man presented to our Ear, Nose, and Throat (ENT) clinic with a three-month history of right otalgia, aural fullness, and hearing loss. He had no other significant past medical or surgical history. On otoscopic examination, a rounded red mass was observed completely filling the right external auditory canal and obscuring the view of the tympanic membrane. The manipulation of the tumor with a Jobson-Horne probe revealed it as a soft, non-tender, and non-friable mass. The results of tuning fork tests showed that Rinne’s test was negative on the right side, and Weber’s test lateralized to the right side indicating right conductive deafness. Facial nerve function was bilaterally normal. Rest of the ENT and systemic examination indicated insignificant results. Pure tone audiometry confirmed conductive deafness with an air-bone gap of 40 db in the right ear (Fig.1).

**Fig 2:**
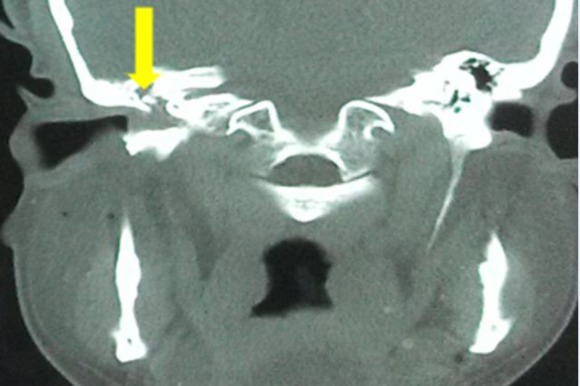
Computed tomography temporal bone (coronal view) showing soft tissue density mass in right tympanic cavity (yellow arrow) extending to the external auditory canal

Based on our clinical assessment, the differential diagnoses included the benign and neoplastic lesions of the external and middle ear. Regarding the extent of the lesion, the performance of a high-resolution computed tomography (CT) scan of the temporal bone indicated the opacification of the right middle ear cavity and the external auditory canal (EAC, Fig.2). Moreover, auditory ossicles and facial canal were intact with no evidence of bony erosion. 

Six days after his initial clinic visit, the patient developed House-Brackmann grade II right-sided facial palsy with complete eye closure on minimal effort as well as mild forehead and oral asymmetry. After the admission of the patient, the intravenous steroid was administered for him. Since the symptoms of pain and acute onset facial palsy are typical symptoms of malignancy, we performed an urgent exploration of the right ear under anesthetic. Intra-operatively, there was a red fleshy mass arising from the middle ear through a large tympanic membrane perforation and filling the right EAC. The biopsy of the lesion was performed and sent for frozen section. The examined sections revealed solid nests of cells composed of small rounded nuclei with evenly dispersed chromatin, inconspicuous nucleoli, and eosinophilic cytoplasm, and there was no invasion of the mastoid cells. All these findings raised the possibility of a benign neoplasm of the middle ear. We subsequently proceeded the treatment procedure with an exploration of the tympanic cavity via a post-auricular approach. The lesion was encasing the malleus and incus; however, there was no obvious bony erosion. In order to achieve the complete excision of the tumor, the malleus and incus were removed after disarticulation at the incudostapedial joint. Furthermore, the tumor did not involve the inner ear and mastoid. After the middle ear mass was completely removed, we noticed edema and hyperemia of the facial nerve from small bony dehiscence in the fallopian canal. In this regard, type III tympanoplasty was performed with temporalis fascia graft placed on the stapes head.

**Fig 3:**
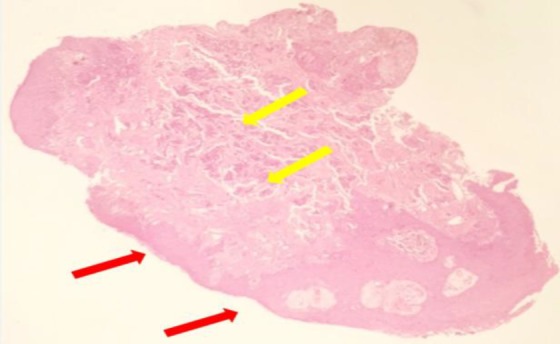
Microscopic examination indicating polypoidal tissue lined by stratified squamous epithelium as a downward extension (red arrows), Subepithelial tissue showing small nests, aggregates, and ribbon-like clusters of small uniform cells (yellow arrows)

The microscopic examination of the tumor indicated round to cuboidal cells with fairly deformed nuclei and absent nucleoli (Fig.3). 

**Fig 4:**
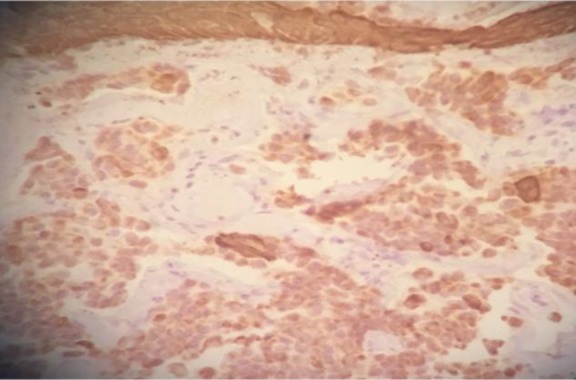
A cross-section through the tumor demonstrating the expression of immunohistochemical marker synaptophysin in favor of neuroendocrine differentiation

Immunohistochemistry was positive for cytokeratins (CKAE1/ AE3, CK7) and synaptophysin (Fig.4). 

Based on these findings, the lesion was diagnosed as NAME. The patient was discharged by a course of oral steroids and antibiotics the day after the surgery. The discussions held about the case in the Multi-Disciplinary Team meeting and it was decided to perform workup to rule out the metastatic potential of the tumor, which was negative. At his 3-week post-operative appointment, his facial nerve function returned to normal (House-Brackmann grade I). There was no evidence of recurrence at a 3-year follow-up.

## Discussion

The neuroendocrine neoplasms of the ear are rare neoplasms accounting for less than 2% of all primary ear tumors (8,9). Among these, NAME is the most prevalent one originating from enterochromaffin cells, which are a part of the amine precursor uptake and decarboxylation system (10). These tumors are considered benign with no bony invasion or metastatic potential (11).

According to the literature, the etiology of NAME is unclear. Middle ear mucosa is derived from the endoderm; however, skin cells with neuroendocrine features are absent in the middle ear cavity. There are two theories explaining the development of such tumors in the middle ear First theory states that an undifferentiated pluripotent endodermal stem cell may still be present in the middle ear mucosal surface. Second describes that the stroma of the middle ear derived from mesoderm and neural crest and the tumor may originate from neural crest-derived stem cell. Regardless of the theory, a metaplastic transformation would be needed within the resident cells of the middle ear for dual population established within these tumors (11).

The neuroendocrine adenoma of the middle ear classified under middle ear glandular neoplasms. Currently, the most widely used classification for middle ear glandular tumors is based on the detection of immunohistochemical markers and presence of metastases proposed by Saliba et al. in 2009 (12). Accordingly, such tumors are categorized into three types. Type I (the most common one with a prevalence of 76%) is NAME, which reveals positive immunohistochemical markers and negative metastases. Type II (20%) is the middle ear adenoma in which immunohistochemical markers and metastases both are negative, and Type III (4%) is the carcinoid tumor indicating positive immunohistochemical markers plus metastases. 

The incidence of the tumor is equal in both male and female patients. The NAME usually occurs in adult patients with the reported age ranges of 20-80 years and mean age of 45 years (11). However, the diagnosis of such tumors is often delayed due to the scarcity of these tumors and their slow growth rate (13). The most common symptoms include unilateral gradual hearing loss, tinnitus, and aural fullness. In addition, some cases suffer from pain and vertigo. Similarly, facial nerve involvement is an atypical finding and can suggest malignant pathology (14). In our case, we needed to exclude a malignant tumor, such as squamous cell carcinoma, as our patient had symptoms of earache and facial palsy. 

The otoscopic examination of NAME usually shows retrotympanic mass. Perforation of the tympanic membrane, with the extension of mass into the external auditory canal (EAC) is a rare finding with only a few cases reported in the literature (1,15). Our case also revealed analogous finding on clinical examination as the tumor was visible on EAC. As reported, the CT scan of NAME showed a well-circumscribed soft tissue mass embedded in auditory ossicles (16), and the bone destruction and invasion of surrounding bones were rare (3,11). Maintz et al. reported signal intensity as the cerebral white matter on T1 and with gray matter on T2 sequences of magnetic resonance imaging (MRI) (17). Differential diagnoses include glomus tumor, middle ear adenoma, squamous papilloma, schwannoma, meningioma, paraganglioma, and cholesteatoma. Given the clinical presentation and radiological findings are non-specific, the final diagnosis could be confirmed by histological and immunohistochemical findings. Histologically, the tumor presented as an unencapsulated tumor with different growth patterns, namely solid, glandular, or trabecular patterns (18). 

Immunohistochemistry plays an important role in differentiating various middle ear glandular neoplasms, including NAME. Therefore, their obtained results from different therapeutic procedures should always be interpreted along with conventional histopathologic findings.

The immunohistochemistry technique involves the application of monoclonal as well as polyclonal antibodies to find out the tissue distribution of an antigen of interest with regard to *de novo* or up-regulated expressions of specific tumor antigens. The advantages of this method outweigh the traditionally used special enzyme staining methods since they detect only a limited number of proteins, enzymes, and tissue structures (19). In NAME, tumor cells show immunoreactivity to a range of cytokeratins and are also positive for neuroendocrine markers, such as chromogranin, enolase, and synaptophysin (7). Our case also showed similar staining pattern in which cells were also strongly positive for cytokeratins and synaptophysin. 

Surgical excision is the treatment of choice. The extent of the procedure is determined by the size and involvement of adjacent structures (20). Smaller lesions confined to middle ear cleft require transcanal tympanotomy (21). On the other hand, a facial recess approach and mastoidectomy are recommended for larger lesions (22). Further larger tumors involving inner ear or mastoid entailed petrosectomy (partial/total) or petromastoidectomy (15). No adjuvant treatment is needed as these tumors are poorly radiosensitive (4,22). In such cases, recurrence is rare after the complete excision of the tumor during the primary surgery. Partial excision is not recommended due to the regrowth and recurrence of symptoms. The recurrence rate is also quite high at 22% in case the ossicular chain is not removed and left intact (11,23). Following the excision of NAME, it is essential to do a long term follow-up. Clinical examination with otoscopy and audiometry would often suffice; however, some authors suggest annual CT or MRI scans in order to eliminate recurrences (24).

Facial palsy secondary to benign middle ear tumors is rare, and the diagnosis of these tumors is a challenging issue given their asymptomatic features. In addition to facial schwannoma, other benign tumors of the middle ear that can cause facial weakness include glomus tumor, adenoma, and haemangioma (25-27). Likewise, there have also been case reports of lipoma and myxoma (28,29). Our patient developed acute facial nerve palsy secondary to NAME. To the best of our knowledge, only Hasan et al. previously described a similar finding of acute facial weakness from NAME in the literature postulating that inflammation without neural invasion may be a causative factor (7). We performed tympanomastoidectomy for total clearance, and facial nerve function recovered fully within a month.

## Conclusion

Acute onset facial palsy due to NAME is extremely rare. For a patient already affected by hearing impairment resulted from middle ear mass, facial weakness can have a significant additional detrimental impact on their wellbeing. We recommend the early complete excision of the tumor, not only as a curative treatment but also restoration of facial function. In this regard, further research is required to have a better understanding of the pathology, management,and long-term outcome of NAME.
